# A two-microRNA signature predicts the progression of male thyroid cancer

**DOI:** 10.1515/biol-2021-0099

**Published:** 2021-09-13

**Authors:** Bingyang Liu, Haihong Shi, Weigang Qiu, Xinquan Wu, Liqiong Li, Wenyi Wu

**Affiliations:** Department of Thyroid and Breast Surgery, The Second Affiliated Hospital of Fujian Medical University, Quanzhou, Fujian 362000, People’s Republic of China

**Keywords:** male thyroid cancer, microRNA signature, tumour progression, miR-451a, miR-16-1-3p

## Abstract

In various cancers, microRNAs (miRNAs) are abnormally expressed, including thyroid cancer (TC). In recent years, the incidence of TC has increased annually around the world. Compared with female patients, male TC patients are more likely to have a postoperative recurrence and lymph node metastasis, and hence need second treatments. However, the molecular biological processes underlying this phenomenon are not understood. Therefore, we collected data on miRNA expression and clinical information of male TC patients from The Cancer Genome Atlas (TCGA) database. Differentially expressed miRNAs were identified between male TC tissues and matched normal tissues. The Kaplan–Meier method, univariate and multivariate Cox regressions, and receiver operating characteristic curve analyses were performed to assess the association between miRNAs and the disease-free survival of male TC patients. Gene Ontology (GO) and the Kyoto Encyclopaedia of Gene and Genome (KEGG) enrichment analyses were then used to explore the function of miRNA target genes. Furthermore, we evaluated the ability of the miRNA biomarker to predict survival in female TC patients. As a result, a total of 118 differentially expressed miRNAs were identified, including 25 upregulated and 93 downregulated miRNAs. Among them, miR-451a and miR-16-1-3p were confirmed to be independent prognostic factors for the disease-free survival rate. The target genes of miR-451a and miR-16-1-3p were identified, and functional analysis showed that these genes were enriched in 25 Go and KEGG accessions, including cell signal transduction, motor adhesion, phagocytosis, regulation of transcription, cell proliferation, angiogenesis, etc. Neither miR-451a and miR-16-1-3p, nor a prediction model based on both miRNAs effectively predicted survival in female TC patients. In conclusion, both miR-451a and miR-16-1-3p may play important roles in the processes of male TC. The two-miRNA signature involving miR-1258 and miR-193a may serve as a novel prognostic biomarker for male TC patients.

## 1 Introduction

Over the past few decades, the incidence of thyroid cancer (TC) has increased substantially in many countries [1]. TC is three times more frequent in women than in men [2], but in multiple studies, the male sex has been shown to be a risk factor for mortality in patients with TC [3,4,5]. Thus far, the studies present conflicting evidence concerning the impact of female hormonal and reproductive processes [6,7,8,9]. One study suggested that the BRAF V600E mutation could be a vital independent risk factor for male TC patients [10]. To date, the role that male sex plays in TC remains unclear and has not been extensively studied. The microRNAs (miRNAs) are a class of endogenous, non-coding small RNAs. These RNAs play crucial roles in many types of cancer by regulating gene expression [11,12]. Although miRNAs themselves do not encode any substances, their abnormal expression may cause tumours through a number of ways [13,14,15]. Many studies have shown that there is a large number of abnormal expressions of miRNAs in the pathogenesis of TC, such as miR-220, miR-22, let-7, and miR-345 [16]. Other miRNAs (e.g., miR-21 and miR-192) are involved in different cell death pathways [17]. However, there is no clear evidence that explains the role of miRNA in the pathogenesis of male TC, particularly with regard to miRNA and patient prognosis. We identified prognostic miRNAs associated with disease-free survival (DFS) time in male TC patients using comprehensive bioinformatic analysis. The miRNA-seq data and clinical information originated from the TCGA database. The functions of miRNA target genes were explored using GO and KEGG enrichment analyses. Finally, we developed a two-miRNA expression signature to predict the DFS rate in male TC patients. Furthermore, we evaluated the ability of the miRNA biomarker to predict survival in female TC cancer patients, and the results showed that neither miR-451a and miR-16-1-3p nor the two-miRNA expression signature were effective in predicting survival in female TC patients.

## 2 Materials and methods

### 2.1 TC miRNA-seq dataset and clinical information

TCGA miRNA-seq data were downloaded from UCSC Xena (https://xenabrowser.net/), and were reported as reads-per-million-miRNA-mapped (RPM) and were normalized by log2(RPM + 1) transformation. The clinical information was obtained from the TCGA database (https://tcga-data.nci.nih.gov) (Table S1). Cases with missing data related to patient sex, patient age at surgery, type of resection, histology, or staging classification or patients who were missing follow-up information were excluded from the study. To rule out the effects of surgical injury, we also excluded individuals whose follow-up or death time was less than seven days. Finally, we established a male TC dataset containing tumour tissues (*n* = 129) and matched normal tissues (*n* = 16). A female TC dataset containing 353 patients was established to verify the unique value of the miRNA biomarkers for male TC patients.

### 2.2 Screening of differentially expressed miRNAs

We used the limma package in R (R version 4.03 and limma version 3.12) to screen the differentially expressed miRNAs between the tumour tissues and normal tissues. The fold change (FC) indicates the degree of differential expression of the miRNA. A log |FC| >2.0 and a *P*-value <0.05 after false discovery rate (FDR) adjustment were established as the cut-off criteria.

### 2.3 Identifying survival-related miRNAs using survival analysis

In the process of survival analysis and the establishment of a prognostic model, we used the survival package (version 3.2-7) in R. In the first step, a total of 129 patients were divided into high- and low-risk groups based on the median expression level of a differentially expressed miRNA. Then, the Kaplan–Meier method with the log-rank test and univariate Cox regression analysis were employed to evaluate the relationship between the expression level of this miRNA and the DFS time of patients. A *P*-value <0.05 was considered statistically significant. The DFS time was defined as the length of time till survival without any signs or symptoms of relapse or metastasis after surgery. Following the same approach, a survival analysis was performed separately for each miRNA. Finally, we identified 10 miRNAs that were closely related to DFS time in male TC patients.

### 2.4 Establishment of a prognostic model based on the expression level of miRNAs

We performed an intersection between differentially expressed miRNAs and miRNAs highly correlated with DFS time. The prognostic value of these miRNAs in the intersection was tested with the Cox proportional hazards model using a multiple logistic regression with forward stepwise logistic regression (LR) selection of variables. Finally, miR-451a and miR-16-1-3p were found to be independent risk factors associated with DSF time in the model. We calculated a risk score for each patient. This score was based on the expression levels of miR-451a and miR-16-1-3p, as determined by the Cox regression coefficient of the miRNA. [18] A total of 129 male TC patients were divided into either the high-risk group (*n* = 64) or the low-risk group (*n* = 65) based on the median value of the risk score. The Kaplan–Meier method was performed to determine the predictive power of the two-miRNA signature for survival prediction. We then performed univariate and multivariate Cox regression analyses to investigate whether the predictive ability of the two-miRNA signature was independent of other clinical features using DFS as the dependent variable and the two-miRNA risk score and other clinical features as the explanatory variables. Clinical features were divided into two groups according to the Eighth American Joint Committee on Cancer (AJCC): age (>60 vs ≤60), histological type (nonpapillary thyroid carcinoma [nonTPC] vs TPC), tumour size (T3–4 vs T1–2), lymph node status (N1 vs N0 and Nx), metastasis (M1 vs M0 and Mx), and pathological stage (S3–4 vs S1–2). A *P*-value <0.05 was considered statistically significant. A student’s *t*-test was applied to assess whether any differences in the two-miRNA expression levels occurred in the two clinical groups. Data are reported as the mean value and standard deviation. A *P*-value <0.05 was set as the cut-off criterion. Receiver operating characteristic (ROC) curves were drawn for variables to determine the optimal cut-off values to predict the highest diagnostic accuracy. Optimal cut-offs of the ROC curve were identified by calculating the Youden index. Overall accuracy was determined using the area under the curve (AUC). We divided 129 patients into a high-risk group (*n* = 13) and a low-risk group (*n* = 116) based on the optimal cut-off value. The DFS times of the two groups were compared using Kaplan–Meier curves. We also calculated the AUC for predicting DFS events within 1, 3, and 5 years.

### 2.5 KEGG pathways and gene ontology analysis

The potential target genes of miR-451a and miR-16-1-3p were predicted using TargetScan 7.2 [19] and miRDB [20,21]. The intersection of the target genes from the 2 databases was put into a gene list for each miRNA. Venn diagrams based on the gene list were constructed using Bioinformatics and Evolutionary Genomics tools (http://bioinformatics.psb.ugent.be/webtools/Venn/). We also analysed the overlapping genes using the DAVID database (version 6.8) (https://david.ncifcrf.gov/) [21,22]. GO and KEGG pathway enrichment analyses were performed to determine the biological processes and pathways involving the target genes. The cut-off criteria for pathway enrichment analyses were *P* < 0.05 and gene counts of ≥5.

### 2.6 Validation of the miRNA biomarkers in female TC patients

The Kaplan–Meier method was employed to evaluate the relationship between the miRNA biomarkers and the DFS time of female TC patients. The miRNA biomarkers contained the expression level of miR-451a, the expression level of miR-16-1-3p, and a two-miRNA signature based on both the miRNAs. A total of 353 female patients were divided into either the high-expression/risk group or the low-expression/risk group based on the median value of the gene expression/risk score.

## 3 Results

### 3.1 Identification of differentially expressed miRNAs in male TC tissues compared with normal thyroid tissues

**Figure 1:**
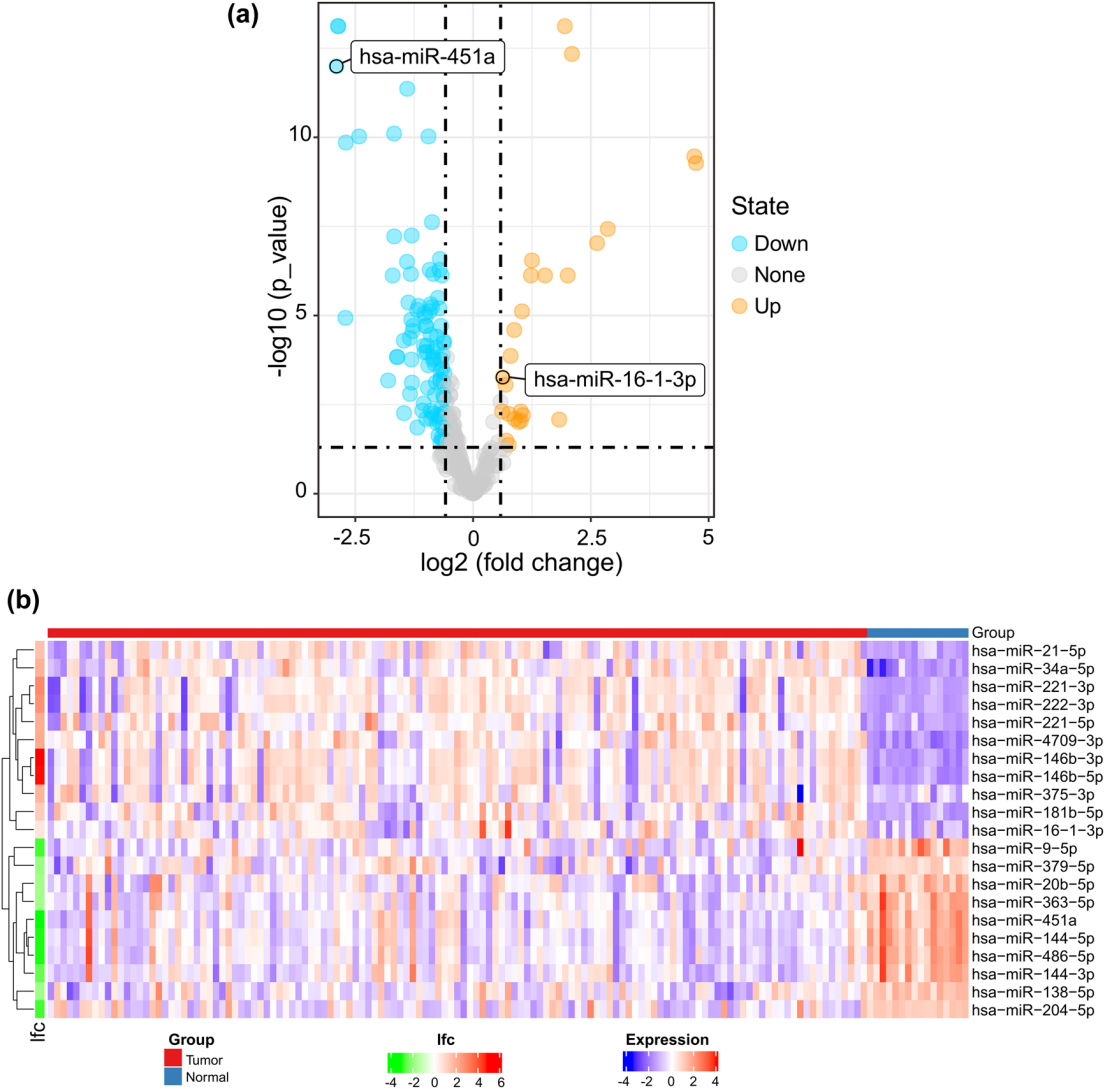
(a) Volcano plot of differentially expressed miRNAs. Yellow dots represent upregulated miRNAs, and blue dots represent downregulated miRNAs. (b) The heat map shows the top 10 upregulated and top 10 downregulated differentially expressed miRNAs. miR-451a and miR-16-1-3p are included in this heat map. A red box represents upregulated expression, whereas a blue box represents downregulated expression. For log fold change (lfc), red represents upregulated expression, whereas green represents downregulated expression; the colour scale for lfc ranges from −4 (green) to 6 (red), and the *x*-axis above the plot indicates grouping of samples in which red is the tumour tissue group and blue is the normal tissue group.

A total of 118 differentially expressed miRNAs were identified, including 25 upregulated and 93 downregulated miRNAs (Figure 1a). The heat map represents the top 10 upregulated and top 10 downregulated differentially expressed miRNAs between tumour tissues and normal tissues; miR-451a and miR-16-1-3p are included in this heat map (Figure 1b).

### 3.2 Identification of miRNAs associated with DFS time in male TC patients

**Figure 2:**
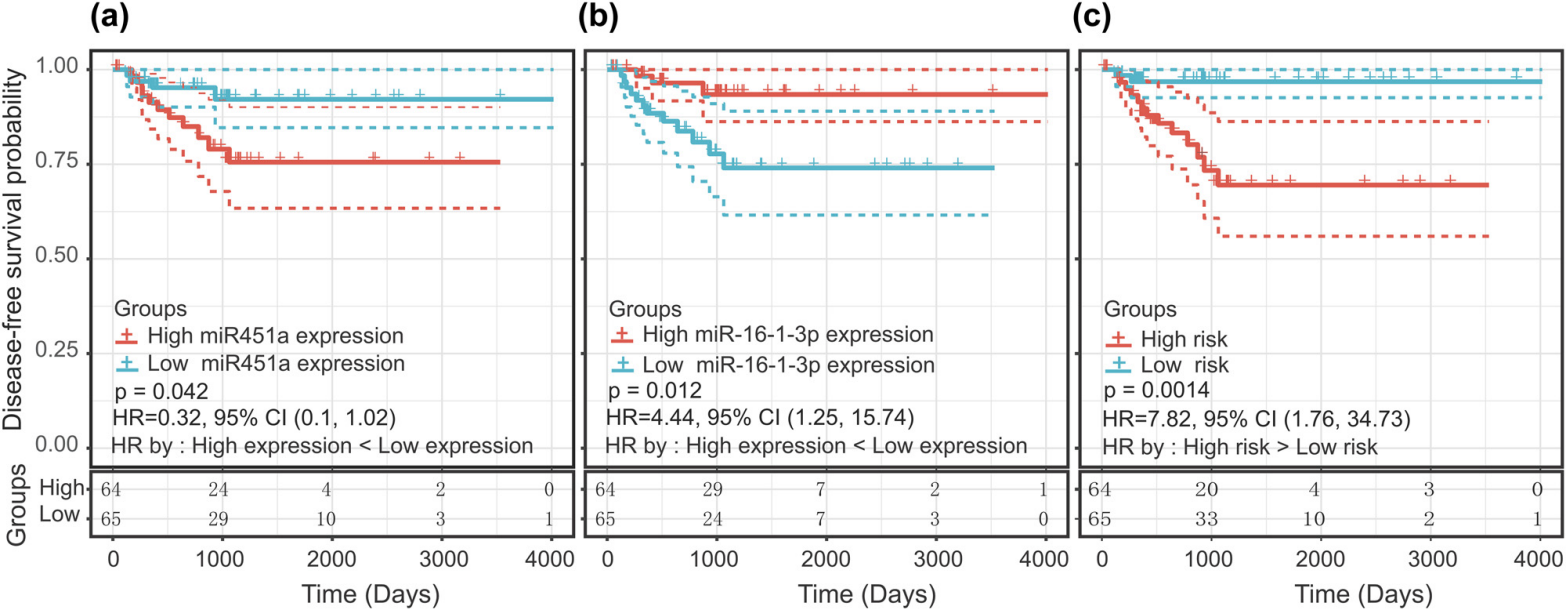
Kaplan–Meier curve with the log-rank test for miRNAs and the miRNA signature. (a) miR-16-1-3p, (b) miR-451a, and (c) two-miRNA signature.

A total of 10 miRNAs were identified as significantly associated with the DFS time of male TC patients: miR-16-1-3p, miR-1307-5p, miR-486, miR-451a, miR-145-3p, miR-139-3p, miR-143-3p, miR-708-5p, miR-582-3p, and miR-141-5p. The intersection between the 10 miRNAs associated with DFS time and the 118 differentially expressed miRNAs revealed 8 miRNAs: miR-16-1-3p, miR-1307-5p, miR-486, miR-451a, miR-145-3p, miR-139-3p, miR-143-3p, and miR-708-5p. These 8 miRNAs were further screened using the multivariate Cox proportional hazards model. Finally, two miRNAs were considered as independent risk factors related to the DFS time of male TC patients (Figure 2a and b). Of these, miR-451a was a protective factor (*B* = −0.714, relative risk [RR] = 0.489, *P* < 0.01, and 95% confidence interval [CI], 1.82–9.01), whereas miR-16-1-3p was a risk factor (*B* = 1.397, RR = 4.045, *P* < 0.01, and 95% CI, 0.30–0.79). The *B* value was the miRNA regression coefficient in the multivariate Cox proportional hazards model. A formula to calculate the risk score for every patient was derived based on his/her individual two-miRNA expression levels weighted by the miRNA regression coefficients as follows: risk score = 1.397 × (expression level of miR-16-1-3p) − 0.714 × (expression level of miR-451a). The patients were divided into low-risk groups (*n* = 65) and high-risk groups (*n* = 64) based on the median risk score (−2.87). Compared with patients in the low-risk group, patients in the high-risk group had a shorter DFS time (hazard ratio [HR] = 7.82; 95% CI, 1.76–34.73; *P* < 0.01) (Figure 2c). In addition, the t-test results showed that miR-451a and miR-16-1-3p were not differentially expressed between any groups of clinical variables (Table 1), which ruled out an association between the expression level of the two miRNAs and clinical groups and allowed for co-linearity between variables to be excluded.

**Table 1:** Associations between the two miRNAs and clinical features

Variables	Numbers	miR-451a	*P*-value	miR-16-1-3p	*P*-value
Patient age at diagnosis					
≤60	89	9.22 ± 1.23	0.98	2.54 ± 0.59	0.33
>60	40	9.23 ± 1.49		2.66 ± 0.64	
Clinical stage（AJCC）					
SI–II	79	9.39 ± 1.28	0.07	2.53 ± 0.57	0.30
SIII–IV	50	8.96 ± 1.32		2.65 ± 0.66	
T stage (AJCC)					
T1–2	69	9.36 ± 1.42	0.20	2.52 ± 0.50	0.22
T3–4	60	9.06 ± 1.16		2.65 ± 0.70	
N stage (AJCC)					
N0 & Nx	60	9.20 ± 1.38	0.87	2.54 ± 0.58	0.47
N1	69	9.24 ± 1.26		2.61 ± 0.63	
M stage (AJCC)					
M0 & Mx	126	9.25 ± 1.31	0.19	2.57 ± 0.61	0.38
M1	3	8.25 ± 1.22		2.88 ± 0.56	
Histologic type					
TPC	125	9.21 ± 1.32	0.48	2.57 ± 0.60	0.12
Non TPC	4	9.68 ± 0.82		2.81 ± 0.80	
Tumour location					
One lobe	97	9.15 ± 1.26	0.32	2.63 ± 0.55	0.12
More than one lobe	32	9.42 ± 1.46		2.43 ± 0.73	

**Figure 3:**
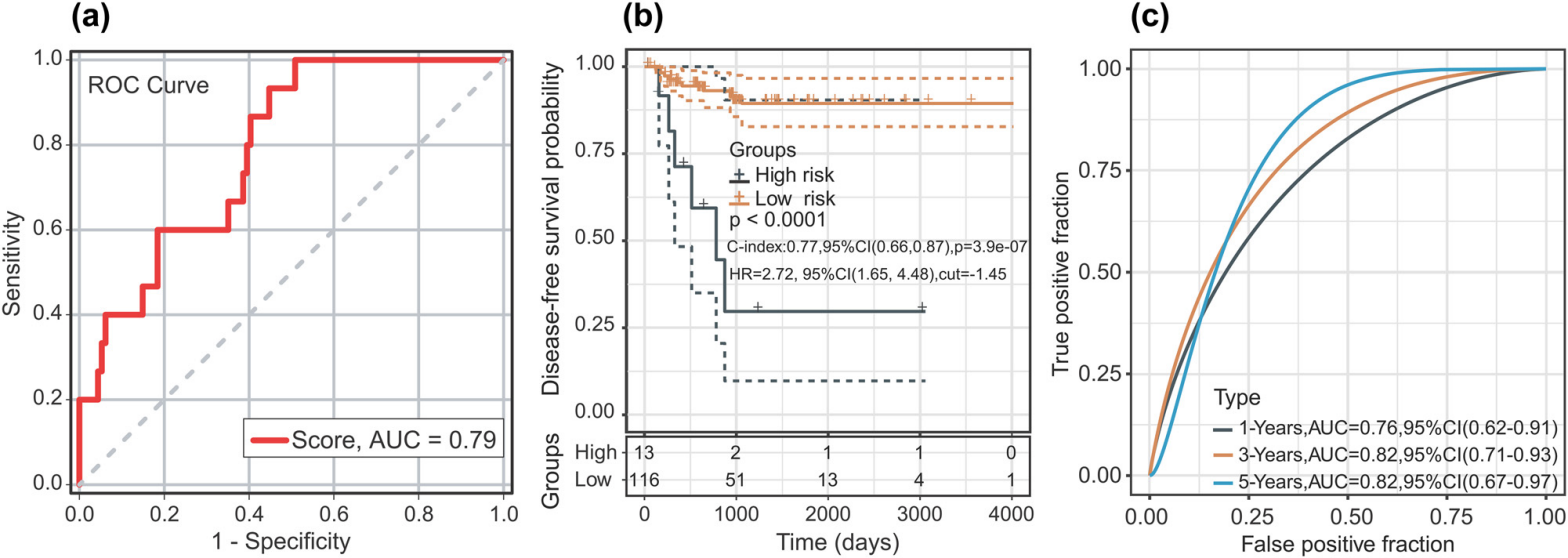
(a) ROC curve of sample for male TC patients. (b) Kaplan–Meier curve after optimal cut-off value; 13 patients were placed into the high-risk group and 116 patients were placed in the low-risk group (optimal cut-off value = −1.45). (c) The AUC curve of the research subject for 1, 3, and 5 years.

According to the ROC analysis, the optimal cut-off risk score for DFS was −3.13 (AUC = 0.789; *P* < 0.01; and 95% CI 0.69 and 0.89). The sensitivity and specificity were 100% (95% CI: 0.78 and 0.99) and 49.12% (95% CI: 0.40 and 0.59), respectively, at the best cut-off point (Figure 3a). The patients were divided into low-risk groups (*n* = 116) and high-risk groups (*n* = 13) based on the best cut-off point. Compared with the patients in the low-risk group, patients in the high-risk group had a shorter DFS time (HR = 2.72; 95% CI, 1.65–4.48; *P* < 0.01) (Figure 3b). We evaluated the ROC curve of 1-, 3-, and 5-year DFS, and the ROC curves demonstrated that the two-miRNA signature harboured a promising ability to predict DFS (1-year AUC = 0.76, 3-year AUC = 0.82, and 5-year AUC = 0.82) (Figure 3c). In the univariate analysis, pathological stage (HR = 4.579; *P* = 0.009; and 95% CI, 1.458–14.386) and the two-miRNA signature (HR = 7.823; *P* = 0.007; and 95% CI, 1.762–34.728) were associated with DFS time in patients with TC. In the multivariate analysis, the two-miRNA signature (HR = 6.937; *P* = 0.015; and 95% CI, 1.466–32.819) was the only independent risk factor for the DFS time of male TC patients (Table 2).

**Table 2:** Univariate and multivariate Cox regression analyses in male TC patients

	Univariate analysis		Multivariate analysis	
	*P* value	HR (95% CI)	*P* value	HR (95% CI)
Age (>60 vs ≤60)	0.071	2.551 (0.923–7.044)	0.928	0.943 (0.266–3.339)
Histological type (NON TPC vs TPC)	0.617	0.047 (0.001–7444.315)	0.988	0.001 (0.001–∞)
Tumour size (T3–4 vs T1–2)	0.056	3.054 (0.972–9.595)	0.552	1.481 (0.406–5.406)
Lymph node status (N1 vs N0 & Nx)	0.871	0.919 (0.332–2.538)	0.447	0.637 (0.201–2.034)
Metastasis (M1 vs M0 & Mx)	0.199	3.784 (0.497–28.819)	0.629	1.717 (0.192–15.361)
Pathological stage (S3–4 vs S1–2)	0.009	4.579 (1.458–14.386)	0.098	3.666 (0.787–17.072)
two-miRNA signature (high risk vs low risk)	0.007	7.823 (1.762–34.728)	0.015	6.937 (1.466–32.819)

### 3.3 KEGG and GO analyses of target genes of the miRNAs in the two-miRNA signature

**Figure 4:**
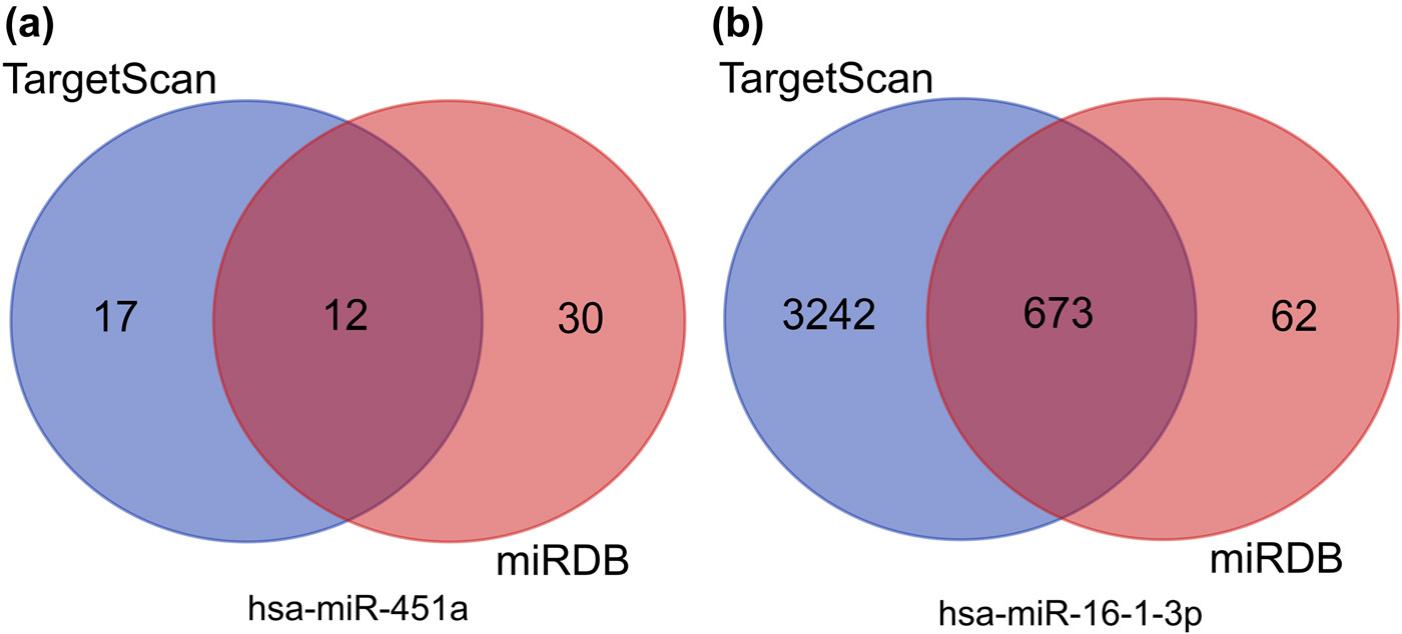
Venn diagrams showing the overlap of target genes that were predicted using the TargetScan and miRDB online tools. (a) hsa-miR-451a and (b) hsa-miR-16-1-3p.

**Figure 5:**
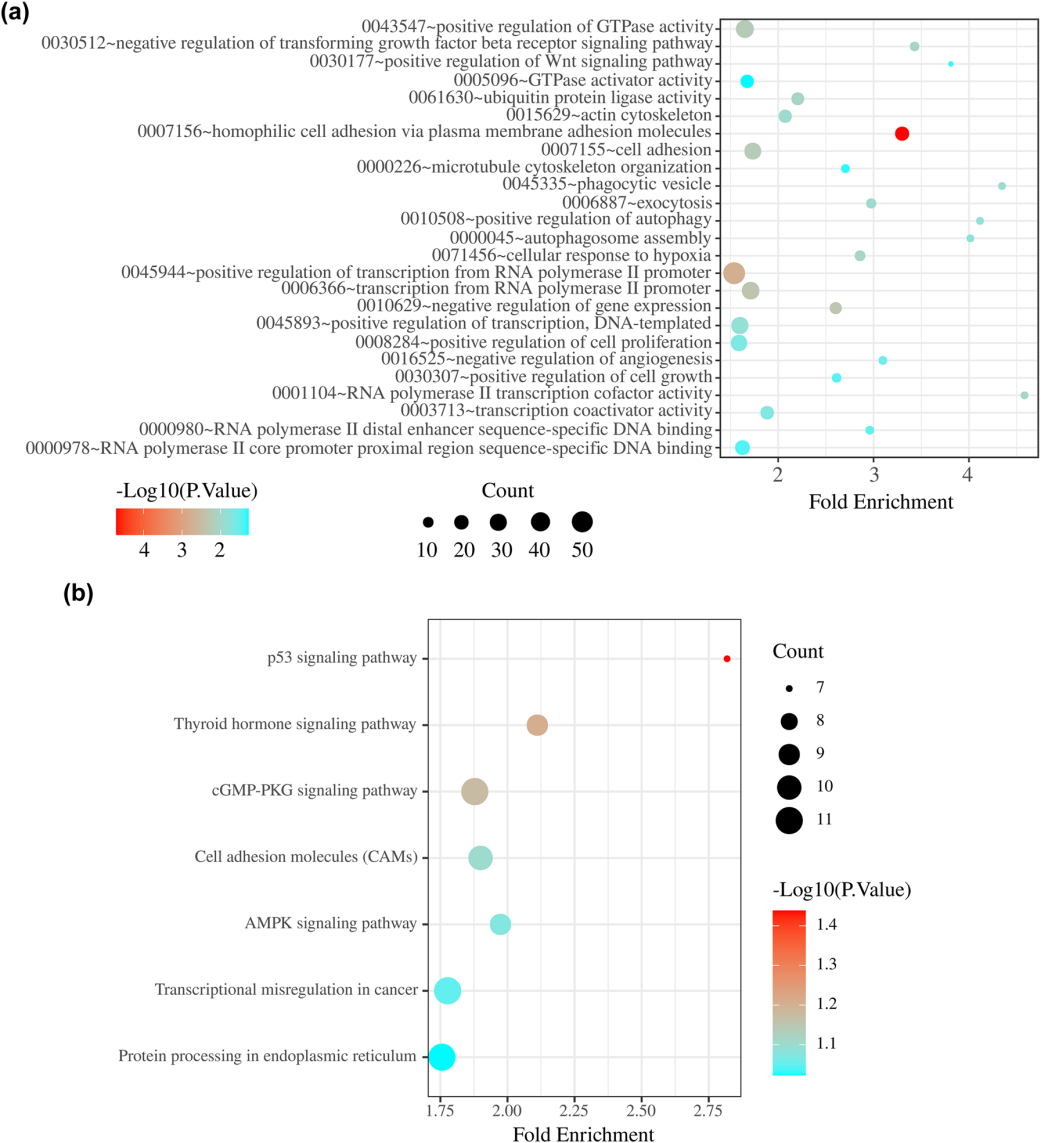
(a) Gene Ontology analysis of two-miRNA signature target genes. (b) KEGG pathway analysis of two-miRNA signature target genes.

A total of 12 overlapping target genes of miR-451a and 673 overlapping target genes of miR-16-1-3p were identified (Figure 4a and b). GO enrichment analysis revealed that these overlapping genes were mainly enriched in cell signal transduction, motor adhesion, phagocytosis, regulation of transcription, cell proliferation, and angiogenesis (Figure 5a). KEGG enrichment analysis revealed significant enrichment for seven KEGG signalling pathways: p53 signalling pathway, thyroid hormone signalling pathway, cGMP-PKG signalling pathway, cell adhesion molecules, AMPK signalling pathway, transcriptional misregulation in cancer, and protein processing in endoplasmic reticulum (Figure 5b).

### 3.4 Predictive value of miRNA biomarkers in female TC patients

**Figure 6:**
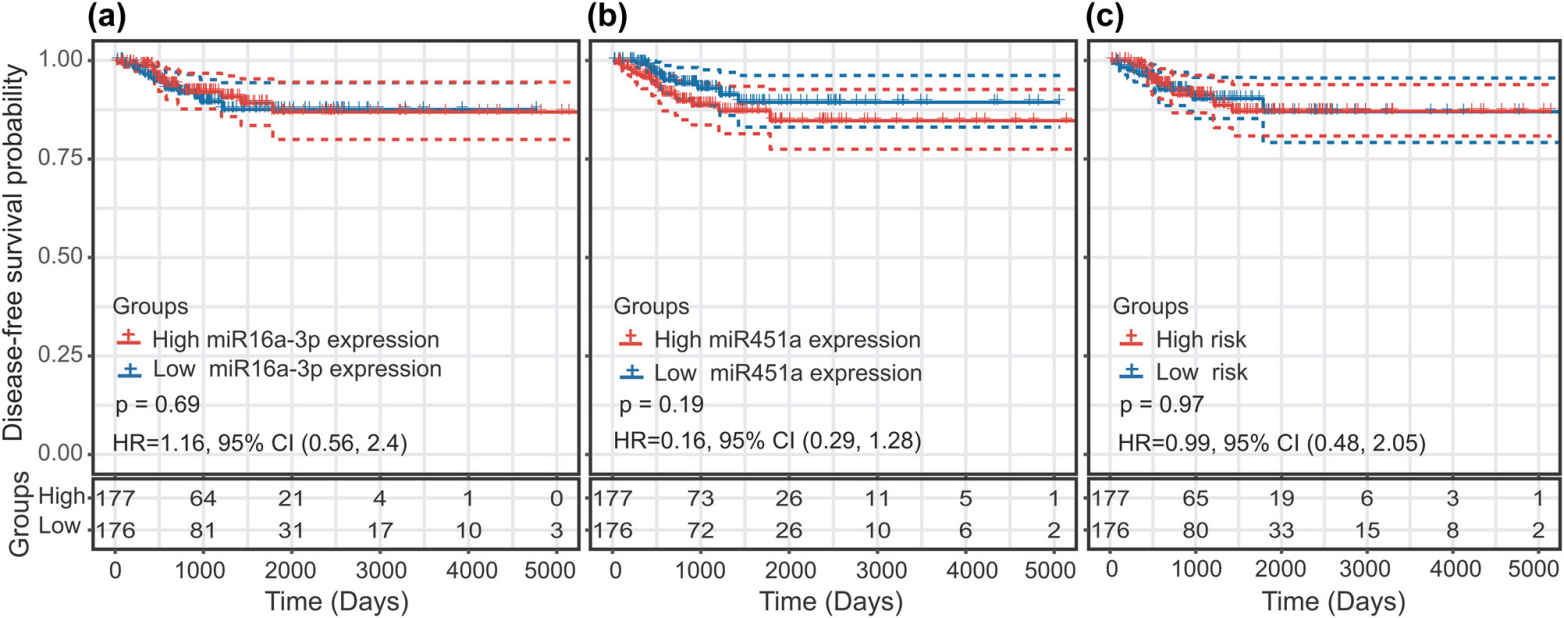
Predictive value of the miRNA biomarkers in female thyroid cancer patients using the Kaplan–Meier curve: (a) miR-16-1-3p, (b) miR-451a, and (c) two-miRNA signature.

In the Kaplan–Meier analysis, there were no significant difference in DFS time between the high and low miR-16-1-3p expression groups (HR = 1.16; *P* = 0.69; and 95% CI, 0.56–2.4) (Figure 6a). Similarly, the difference in the DFS time between the high and low hsa-miR-451a expression groups was not statistically significant (HR = 0.61; *P* = 0.19; and 95% CI, 0.29–1.28) (Figure 6b). There was also no statistically significant difference in DFS time between the high-risk group and the low-risk group (HR = 0.99; *P* = 0.97; and 95% CI, 0.48–2.05) (Figure 6c). Neither miR-451a and miR-16-1-3p nor a prediction model based on both miRNAs were effective in predicting the survival of female TC patients.

## 4 Discussion

In this study, we identified a pathological stage-related two-miRNA signature as a promising predictor of DFS for male TC patients. In the multivariate Cox model, this signature was identified as an independent predictive factor. We evaluated the ability of the miRNA biomarker to predict the survival in female TC patients. The results showed that neither miR-451a and miR-16-1-3p nor a two-miRNA signature based on both miRNAs were effective in predicting the survival in female TC patients. These two miRNAs might play a unique role in male TC development. KEGG and GO analyses revealed that the two-miRNA signature plays crucial roles in cell adhesion, cell motility, cell signalling, transcription control and regulation of gene expression, cell proliferation, angiogenesis, and cellular responses to hypoxia. The above results suggest that the aforementioned two miRNAs are closely related to the biological behaviour of male TC, particularly invasion and metastasis.

The molecule miR-451a has been studied in many types of cancer such as stomach cancer, hepatocellular carcinoma, bladder cancer, colorectal cancer, and basal cell carcinoma. Several studies have suggested that a low expression level of miR-451a positively correlates with the metastatic ability and invasiveness of cancer cells [23,24,25,26,27]. Some studies suggest that miR-451a can be used as a biological marker to predict the risk of recurrence [23,24]. In the field of TC research, experimental data from multiple cell types have confirmed that miR-451a could suppress tumour cell proliferation and invasion by targeting PSMB8 and ZEB1 [28,29]. One study revealed that the expression of miR-451a is downregulated in TC tissues, and lower expression of miR-451a correlates with aggressive clinicopathological features of papillary thyroid carcinoma (PTC). These effects may be related to the AKT/mTOR pathway [30]. All these results indicate that miR-451a might play a protective role in combating cancer in the occurrence and development of TC, which is consistent with our conclusions. miR-451a might be used as a therapeutic target for certain drugs by upregulating the expression of miR-451a to inhibit tumour cell invasiveness.

miR-16 has been extensively studied in other cancer tissues. In ovarian cancer, studies have found that the high expression of miR-16-1-3p is related to the recurrence of ovarian cancer [31]. In a study on cholangiocarcinoma (CCA), miR-16 was one of the largest nodes in the ceRNA regulatory network, which indicates that miR-16 might play an essential role in CCA development [32]. Another study showed that the upregulation of miR-16 can increase the invasiveness of cancer cells through the cell signalling factor pathway in triple-negative breast cancer patients with brain metastases. This process could be related to epithelial-mesenchymal transition [33]. Bladen et al. [34] also suggested that miR-16 is the central factor leading to high invasiveness in sebaceous gland carcinoma. All the above results support the suggestion that miR-16 is a risk factor, which is consistent with our results.

The KEGG pathway analysis showed that the target genes of miR-451a and miR-16-1-3p were involved in the p53 and AMPK signalling pathways. Mutations in p53 have long been noticed in TC [35]. Maroof et al. [36] reported that miRNAs could regulate apoptosis in TC cells by targeting p53. The AMPK signal transduction pathway has also been extensively investigated, and researchers hold that the AMPK signalling pathway may contribute to the initiation, progression, and recurrence of cancer [37,38]. When using AMPK inhibitors to inhibit the AMPK signalling pathway, there is a strong anti-cancer effect in cell induction [39]. Several studies have suggested that activation of the AMPK signalling pathway leads to nuclear translocation of pyruvate kinase M2, which helps cancer cells survive under metabolic stress and promotes cancer cell invasion and metastasis [40,41]. In TC research, Xu et al. [42] found that TMP21 regulates TCP1 cell growth by inducing autophagy, which may lead to activation of the AMPK/mTOR pathway. All these studies provide indirect evidence in support of our conclusion that miR-451a and miR-16-1-3p may influence male TC progression by regulating the activity of the p53 and AMPK pathways.

Transcriptional regulation plays important roles in the processes of eukaryotic gene expression. Transcriptional regulation modifies the gene expression level by altering the efficiency of gene transcription [43]. Studies have proven that the occurrence of cervical cancer is related to transcription failure [44]. Cell experiments have also confirmed that miRNAs can upregulate or downregulate the transcription of target genes in breast cancer by activating cell signalling pathways that promote breast cancer [45]. Many transcription factors can regulate transcription products by regulating the activity of RNA polymerase. Runt-related transcription factor 2 (RUNX2) is an important regulator of osteogenic transcription. This factor was found to be involved in the process of TC calcification and invasiveness. *In vitro* cell experiments found that the enhanced activity of the RUNX2 promoter can enhance the activity of alkaline phosphatase, thereby promoting calcification and the migration and invasion of cancer cells [46]. Recent studies have also shown that cell proliferation, the cell cycle, apoptosis, and autophagy are key techniques to control the development and progression of PTC [47,48]. These findings suggest that miR-451a and miR-16-1-3p may regulate the progression of male TC through the regulation of transcription and cell proliferation.

Cell adhesion and the actin cytoskeleton are closely related to haematogenous metastatic spread of tumour cells [49]. E-cadherin, which plays an important role in normal cell-cell adhesion, was found to be expressed at low levels in TC tumour tissue. This leads to the loss of cell-cell adhesion, allowing TC cell migration [50]. A structural variant of the actin cytoskeleton is an important process in cancer cell metastasis [51]. One study indicated that TC cells are three- to five-fold softer than normal thyroid cells [52]. Angiogenesis also plays a pivotal role in tumour progression [53]. Abnormal angiogenesis has been found in several histopathologic subtypes of TC [54]. This may be related to VEGF-A overexpression in TC cells [55]. In conclusion, the progression of male TC may be affected by miR-451a and miR-16-1-3p via angiogenesis.

## 5 Conclusion

Overall, this study identified two miRNAs that are related to the DFS of male TC patients. These two miRNAs may play an important role in the development of male TC. The two-miRNA signature involving miR-1258 and miR-193a may serve as a novel prognostic biomarker for male TC patients. This is the first study to provide evidence for the relationship between miRNAs and male TC. Our study has some limitations. The results obtained from TGCA need to be verified by *in vitro* cell experiments and large-sample clinical trials, and the molecular mechanisms involving miRNA signatures in male TC also need to be investigated further.
