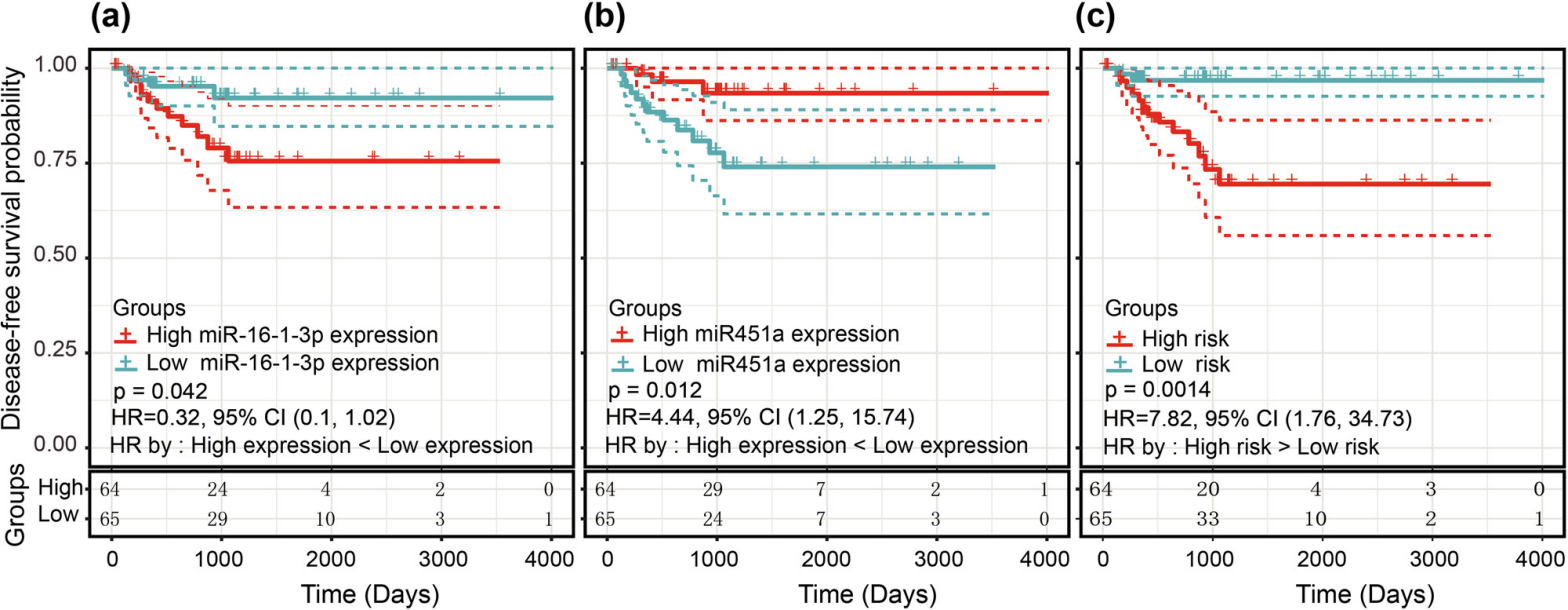# Erratum to “A two-microRNA signature predicts the progression of male thyroid cancer”

**DOI:** 10.1515/biol-2022-0083

**Published:** 2022-09-08

**Authors:** Bingyang Liu, Haihong Shi, Weigang Qiu, Xinquan Wu, Liqiong Li, Wenyi Wu

**Affiliations:** Department of Thyroid and Breast Surgery, The Second Affiliated Hospital of Fujian Medical University, Quanzhou, Fujian 362000, People’s Republic of China

In the published article Liu B, Shi H, Qiu W, Wu X, Li L, Wu W. A two-microRNA signature predicts the progression of male thyroid cancer. Open Life Sci. 2021;16(1):981–91, doi: 10.1515/biol-2021-0099, the authors found some marking errors in Figure 2. In Figure 2a, “miR451a” should be changed to “miR16-1-3p.” In Figure 2b, “miR16-1-3p” should be changed to “miR451a.” The figure legend is correct and does not need to be modified. The authors admit to the error and claim that this is an unintentional mistake that has nothing to do with academic misconduct and does not influence the conclusion of the publication. The authors apologize to the journal readers for the mistake and any inconvenience it caused.

**Figure 2:**